# Artificial Intelligence (Enhanced Super-Resolution Generative Adversarial Network) for Calcium Deblooming in Coronary Computed Tomography Angiography: A Feasibility Study

**DOI:** 10.3390/diagnostics12040991

**Published:** 2022-04-14

**Authors:** Zhonghua Sun, Curtise K. C. Ng

**Affiliations:** 1 Discipline of Medical Radiation Science, Curtin Medical School, Curtin University, P.O. Box U1987, Perth, WA 6845, Australia; curtise.ng@curtin.edu.au; 2 Curtin Health Innovation Research Institute (CHIRI), Faculty of Health Sciences, Curtin University, P.O. Box U1987, Perth, WA 6845, Australia

**Keywords:** assessment, calcification, cardiac computed tomography, coronary artery disease, deep learning, model

## Abstract

Background: The presence of heavy calcification in the coronary artery always presents a challenge for coronary computed tomography angiography (CCTA) in assessing the degree of coronary stenosis due to blooming artifacts associated with calcified plaques. Our study purpose was to use an advanced artificial intelligence (enhanced super-resolution generative adversarial network [ESRGAN]) model to suppress the blooming artifact in CCTA and determine its effect on improving the diagnostic performance of CCTA in calcified plaques. Methods: A total of 184 calcified plaques from 50 patients who underwent both CCTA and invasive coronary angiography (ICA) were analysed with measurements of coronary lumen on the original CCTA, and three sets of ESRGAN-processed images including ESRGAN-high-resolution (ESRGAN-HR), ESRGAN-average and ESRGAN-median with ICA as the reference method for determining sensitivity, specificity, positive predictive value (PPV) and negative predictive value (NPV). Results: ESRGAN-processed images improved the specificity and PPV at all three coronary arteries (LAD-left anterior descending, LCx-left circumflex and RCA-right coronary artery) compared to original CCTA with ESRGAN-median resulting in the highest values being 41.0% (95% confidence interval [CI]: 30%, 52.7%) and 26.9% (95% CI: 22.9%, 31.4%) at LAD; 41.7% (95% CI: 22.1%, 63.4%) and 36.4% (95% CI: 28.9%, 44.5%) at LCx; 55% (95% CI: 38.5%, 70.7%) and 47.1% (95% CI: 38.7%, 55.6%) at RCA; while corresponding values for original CCTA were 21.8% (95% CI: 13.2%, 32.6%) and 22.8% (95% CI: 20.8%, 24.9%); 12.5% (95% CI: 2.6%, 32.4%) and 27.6% (95% CI: 24.7%, 30.7%); 17.5% (95% CI: 7.3%, 32.8%) and 32.7% (95% CI: 29.6%, 35.9%) at LAD, LCx and RCA, respectively. There was no significant effect on sensitivity and NPV between the original CCTA and ESRGAN-processed images at all three coronary arteries. The area under the receiver operating characteristic curve was the highest with ESRGAN-median images at the RCA level with values being 0.76 (95% CI: 0.64, 0.89), 0.81 (95% CI: 0.69, 0.93), 0.82 (95% CI: 0.71, 0.94) and 0.86 (95% CI: 0.76, 0.96) corresponding to original CCTA and ESRGAN-HR, average and median images, respectively. Conclusions: This feasibility study shows the potential value of ESRGAN-processed images in improving the diagnostic value of CCTA for patients with calcified plaques.

## 1. Introduction

Coronary artery calcium is considered a powerful tool for the prediction of coronary artery disease (CAD) risk when compared to traditional risk factors. Its use is integrated into the recent guidelines for further risk assessment, and its wider use in patient screening is suggested to allow for a more personalised risk assessment [1,2]. However, the blooming artifact in coronary computed tomography angiography (CCTA) resulting from radiodense calcium deposits within the coronary plaques affects the determination of an accurate coronary artery calcium score (CS) [3,4]. The blooming artifact in CCTA occurs when a high-density calcified plaque and a much-lower-density vessel are contained in one image voxel, causing an average value of attenuations of these two used to represent them. This makes the calcified plaque on CCTA images appear larger than its true size because of the voxel value close to that of high-density materials. Hence, the blooming artifact can be reduced by improving the image’s spatial resolution [5]. Previous studies have shown that improvement of CCTA spatial resolution through image processing (sharpening) technique can suppress the blooming artifact [3,4,6,7]. However, the traditional (rule-based) deblooming algorithms are unable to address the common problem of image sharpening technique which is noise increase, leading to diagnostic accuracy reduction on a per patient basis [6,7].

Recently, artificial intelligence (AI)-based (specifically deep learning [DL]-based) image reconstruction and synthesis has become an active research area in radiology [8,9]. Commercially available DL (convolutional neural network [CNN])-based computed tomography (CT) image reconstruction kernels such as Canon Medical Systems Advanced Intelligent Clear-IQ Engine (AiCE) and General Electric Healthcare TrueFidelity have also become available on the CT systems [10,11,12,13]. Usually, supervised learning with pairs of low-dose (noisy) and high-dose (quality) CT images as training image data is used to develop these CNN kernels for noise suppression [10,12,13,14]. Studies have shown that these CNN kernels are able to reduce the CCTA image noise and improve image quality [14,15]. Nonetheless, apart from the CNN approach, use of generative adversarial network (GAN) has also become popular in radiology over the past few years [9]. For example, Wolterink et al. demonstrated the feasibility of using a GAN model (trained by pairs of low-dose and routine-dose cardiac CT images via the supervised learning) for noise reduction, enabling the CS determination on low-dose cardiac CT images [16].

However, other than denoising, the GAN approach is also useful for increasing image spatial resolution. In 2016, Ledig et al. reported a seminal work of super-resolution GAN (SRGAN) which was able to generate high-resolution photo-realistic natural images (4 times upscaling) based on low-resolution input images [17]. Subsequently, Wang et al. developed an enhanced SRGAN (ESRGAN) based on the seminal work by Ledig et al. resulting in a first-place award in Perceptual Image Restoration and Manipulation (PIRM) challenge on perceptual super-resolution at the European Conference on Computer Vision (ECCV) 2018. Their study has demonstrated that the ESRGAN is capable of generating high-resolution realistic images with unwanted noise removal [18]. Since the suppression of blooming artifacts for accurate CS determination requires low-noise and high-resolution CCTA images, the ESRGAN appears as an appropriate approach for calcium deblooming in CCTA, which warrants further investigation [6,7,18]. The purpose of this study was to determine the feasibility of using the ESRGAN for calcium deblooming in CCTA. We hypothesised that the use of ESRGAN would lead to improvement in assessing coronary lumen stenosis despite presence of heavy calcification in the coronary arteries, hence contributing to the increased accuracy of CCTA in the diagnosis of calcified plaques. In this study, we compared ESRGAN-processed images with original CCTA images in terms of assessing the degree of coronary stenosis with invasive coronary angiography (ICA) as the reference method, thus allowing us to determine the impact of ESRGAN model on CCTA performance.

## 2. Materials and Methods

### 2.1. CCTA and Invasive Coronary Angiography Images

Anonymised CCTA and ICA (reference) image datasets in Digital Imaging and Communications in Medicine (DICOM) format of 50 adult patients who undertook the CCTA in 2014 with calcified plaques identified in at least one coronary artery segment on the CCTA and subsequent ICA performed for coronary artery stenosis diagnosis were collected [19]. Due to the retrospective study nature, institutional review board approval was waived and informed consent was not required since CCTA and ICA examinations were performed as part of diagnostic procedures. Table 1 shows the patient characteristics.

The CCTA images were acquired by a first-generation dual-source CT (Somatom Definition, Siemens Healthcare, Forchheim, Germany) (19 patients, 38%), a second-generation dual-source CT (Somatom Definition Flash, Siemens Healthcare, Forchheim, Germany) (19 patients, 38%), and a 640-slice CT (Toshiba Aquilion ONE, Toshiba, Otawara, Japan) (12 patients, 24%). The followings were their reconstruction slice thicknesses and intervals: 0.6–0.75 mm and 0.5–0.6 mm (first- and second-generation dual-source CT), and 0.5 mm and 0.25 mm (640-slice CT), respectively. Details of the imaging protocols were reported previously. Our previous study showed that the use of CCTA images acquired by various systems did not have any significant difference in the diagnostic value, and hence, these CCTA images were considered suitable for this study [19].

### 2.2. Deblooming in CCTA

The calcium deblooming in CCTA was performed through four times upscaling the size of the collected CCTA images from 512 × 512 pixels to 2048 × 2048 pixels with the use of the open-source ESRGAN model by Wang et al. [18]. Its source code in PyTorch v1.0.0 (Meta Platforms, Inc., Menlo Park, CA, USA) was available at https://github.com/xinntao/ESRGAN, accessed on 1 February 2022. Unlike the traditional CNN approach (which was also used in the commercial CT image reconstruction kernel development) for super-resolution (SRCNN) [10,11,12,13,20], training of two CNN-based models (generator and discriminator) was required for the SRGAN development. However, Ledig et al. overcame the technological challenge in 2016 and developed the SRGAN model based on the CNN (Visual Geometry Group [VGG] network). The high-resolution images generated by the SRGAN were able to show high-frequency details and looked more realistic than those produced by the SRCNN, which appeared overly smoothed [17]. Wang et al. [18] further advanced Ledig et al.’s [17] SRGAN model as the ESRGAN to generate more realistic high-resolution images through the following strategies.

1. Generator: Removal of the batch normalization layers from the SRGAN with the addition of residual-in-residual dense block layers to facilitate training of a deeper model.
2. Discriminator: Implementation of the relativistic average discriminator to determine whether an image was more realistic rather than “real” or “fake” noted in the SRGAN.
3. Loss function: Determination of the perceptual loss based on the VGG features before its activation rather than after the activation employed in the SRGAN for better monitoring of image brightness and texture.

The ESRGAN model was trained by three publicly available image datasets, DIV2K with 800 images (https://data.vision.ee.ethz.ch/cvl/DIV2K/, accessed on 1 February 2022), Flickr2K with 2650 images (http://cv.snu.ac.kr/research/EDSR/Flickr2K.tar, accessed on 1 February 2022), and OutdoorSceneTraining (OST) with 10,324 images (http://mmlab.ie.cuhk.edu.hk/projects/SFTGAN/, accessed on 1 February 2022). These datasets provided a great variety of colour photos with different resolutions (including 2k resolution) and scenes/objects such as mountains, buildings, animals and humans but unrelated to medical imaging. To increase the variety, these images were flipped and rotated randomly for training. Details of the ESRGAN are available from the article by Wang et al. [18].

Wang et al.’s [18] ESRGAN model was deployed on the free Kaggle platform (Google LLC, Mountain View, CA, USA) with one NVidia K80 graphics processing unit (Santa Clara, CA, USA) to generate the high-resolution images (ESRGAN-HR) (2048 × 2048 pixels) based on the corresponding original resolution images (512 × 512 pixels) of the 50 CCTA datasets collected. About 10 min was required to generate the high-resolution images for each dataset with hundreds of slices. To further suppress the image noise for assessing the coronary lumen stenosis, the open-source image processing program, ImageJ (v1.52a, National Institutes of Health, Bethesda, MD, USA) was used to carry out average (ESRGAN-Average) and median (pixel) binning (ESRGAN-Median) to reduce the high-resolution image matrix size back to the original (512 × 512 pixels) and generate two other CCTA datasets (average and median binned datasets) [21,22]. Each dataset had 50 CCTA cases with the images of the size, 512 × 512 pixels.

### 2.3. CCTA Measurements

Original CT data and ESRGAN-processed images were transferred to a separate workstation equipped with Analyze V 12.0 (AnalyzeDirect, Inc., Lexana, KS, USA) for measurements of coronary stenosis caused by calcified plaques. The minimal lumen diameter (MLD) of three main coronary arteries, namely left anterior descending (LAD), left circumflex (LCx) and right coronary artery (RCA) was measured at each calcified plaque lesion using the same approach as described in our previous studies [4,19]. Measurements were performed by one researcher (with more than 20 years of experience in interpreting CCTA images), with each measurement repeated three times and the mean value was taken as the final.

### 2.4. ICA Measurements

ICA as a reference method to determine the degree of coronary stenosis was performed in all these patients following the standard femoral or radial approach. The MLD was measured by the same observer in angiographic projections where calcified plaques caused the most severe narrowing of the above-mentioned three main coronary arteries. The interval between CCTA and ICA measurements was 4 weeks to avoid potential influence or biased opinion on measurement accuracy. Similarly, three measurements of the same calcified lesion were obtained, with the mean value averaged.

### 2.5. Reduction of Blooming Artifact by ESRGAN

To study the impact of ESRGAN on reducing the blooming artifact with resultant improvement in coronary lumen visualisation and assessment, we also compared the percentage of reduction of MLD in these measurements by subtracting the MLD from original CCTA images to ESRGAN-processed images, with differences divided by the MLD from the original CCTA images. This allowed us to determine the diagnostic value of ESRGAN-processed images for assessment of calcified plaques, in particular the reduction of false-positive rates caused by severe calcification in the coronary arteries as opposed to those from the original CCTA images.

### 2.6. Statistical Analysis

Statistical analyses were performed using SPSS 27.0 (International Business Machines Corporation, New York, NY, USA). Continuous variables were expressed as the mean ± standard deviation, while categorical variables were presented as percentages. Sensitivity, specificity, positive predictive value (PPV), negative predictive value (NPV), positive likelihood ratio (PLR) and negative likelihood ratio (NLR) for the detection or exclusion of significant stenosis (>50%) on CCTA were calculated and compared between original CCTA and ESRGAN-processed images with ICA as the reference. Receiver operating characteristic (ROC) analysis, including area under the ROC curve (AUC), was employed for comparison of diagnostic performance between these imaging methods. Three-way analysis of variance (ANOVA) with pairwise comparisons post-hoc tests was performed to compare differences among these measurements from 5 datasets (ICA, original CCTA, ESRGAN-HR, ESRGAN-Average and ESRGAN-Median) with *p*-value of <0.05 indicating statistically significant.

## 3. Results

A total of 184 calcified plaques from 150 coronary arteries of the 50 patients were identified for inclusion in the analysis. Table 1 shows details of the plaque distribution in these three coronary arteries. All LAD branches had at least one calcified plaque, with most of them having 1–3 plaques, while all LCx had less than three plaques in the branches. In three cases, RCA had more than five plaques, with six, eight and nine plaques detected in each patient, respectively.

**Figure 1:**
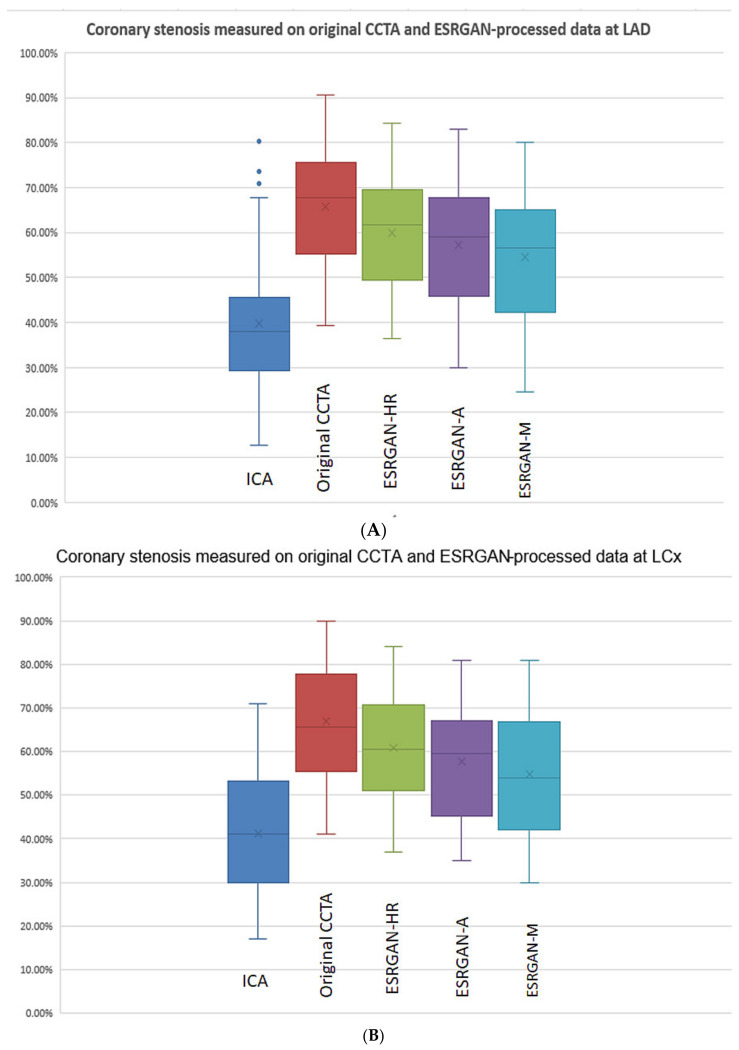

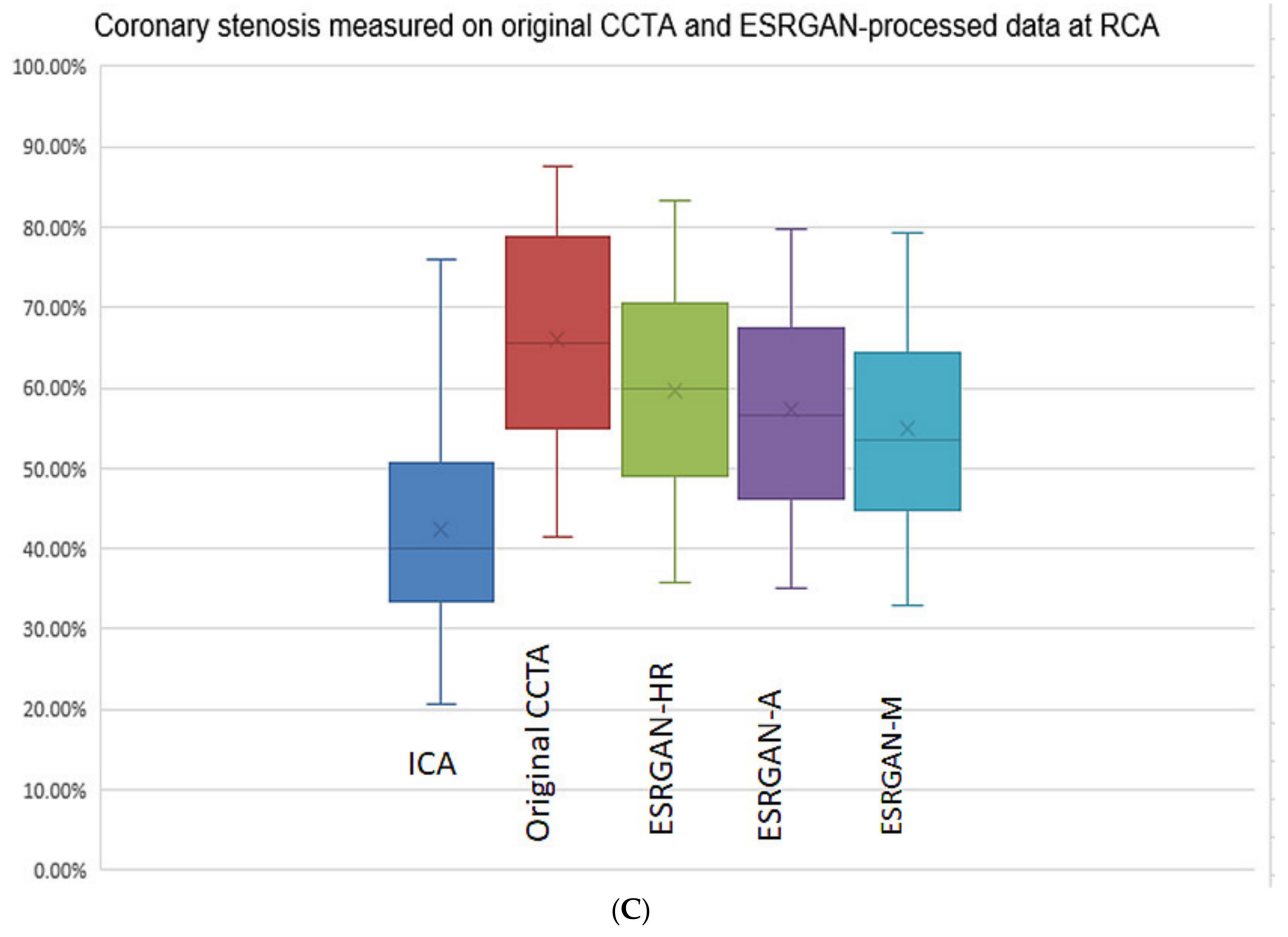
Boxplot showing the comparison of degree of coronary stenosis at LAD, LCx and RCA between original CCTA and ESRGAN-processed data with invasive coronary angiography as the reference. Although original CCTA and ESRGAN-processed images significantly overestimated the lumen stenosis than ICA, ESRGAN-Median images show better improvement than ESRGAN-HR and Average at these three coronary arteries levels (**A**–**C**). A-average; CCTA-coronary computed tomography angiography; ESRGAN-enhanced super-resolution generative adversarial network; HR-high-resolution; ICA-invasive coronary angiography; LAD-left anterior descending; LCx-left circumflex; M-median; RCA-right coronary artery.

**Figure 2:**
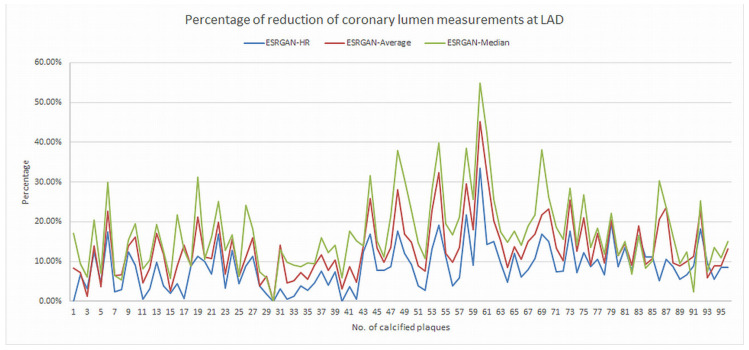

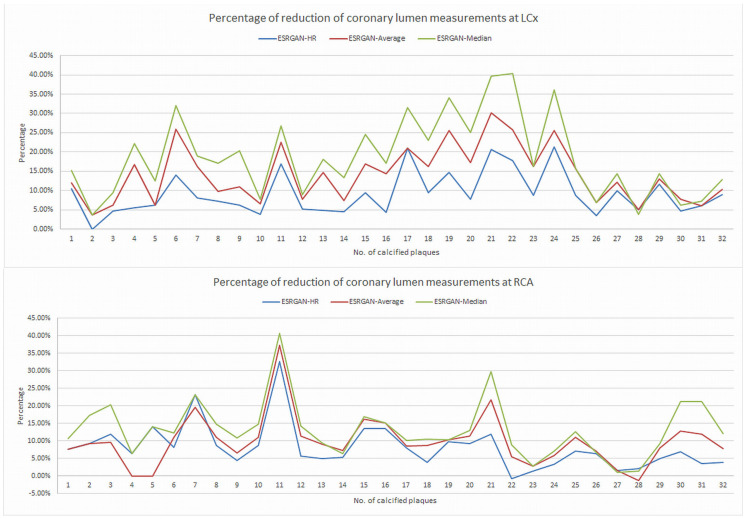
Graphs showing the comparison of measurement differences in coronary lumen stenosis between ESRGAN-processed and original CCTA images in terms of percentage reduction at LAD, LCx and RCA. ESRGAN-Median processed images lead to the highest reduction than the ESRGAN-HR and Average. CCTA-coronary computed tomography angiography; ESRGAN-enhanced super-resolution generative adversarial network; HR-high-resolution; LAD-left anterior descending; LCx-left circumflex; No.-number; RCA-right coronary artery.

There were significant differences in the MLD measurements at these coronary plaques between original, ESRGAN-processed images, and ICA, with significant overestimation of the lumen stenosis due to the presence of calcified plaques on CCTA images (*p* < 0.001 for all comparisons: Original and ESRGAN-HR/ESRGAN-Average/ESRGAN-Median vs. ICA) (Figure 1). ESRGAN-processed images improved the lumen assessment with significant differences compared to the original CCTA images (*p* < 0.001 for all comparisons, ESRGAN-HR/ESRGAN-Average/ESRGAN-Median vs. original CCTA). There were no significant differences in the MLD measurements among the ESRGAN-processed images (*p* > 0.05 for all comparisons at three coronary arteries), although ESRGAN-Median processed images resulted in better improvement or reduction of blooming artifact than the other two ESRGAN-processed datasets. Figure 2 shows the impact of ESRGAN-processed images on the assessment of MLD at these three coronary arteries in terms of the percentage of reduction when compared to that measured on original CCTA images. There were significant differences regarding the degree of reduction of blooming artifacts between comparisons among these ESRGAN-processed images (*p* < 0.05). As shown in Figure 2, ESRGAN-Median processed images produced the highest reduction in all the coronary arteries, with mean percentages of reduction being: 8.62 ± 5.74%, 13.2 ± 7.55% and 17.15 ± 9.59% at LAD; 9.11 ± 5.52%, 14.14 ± 7.25% and 18.61 ± 10.34% at LCx; 9.92 ± 6.12%, 12.96 ± 7.62% and 16.86 ± 8.89% at RCA; corresponding to ESRGAN-HR, ESRGAN-Average and ESRGAN-Median images, respectively.

**Figure 3:**
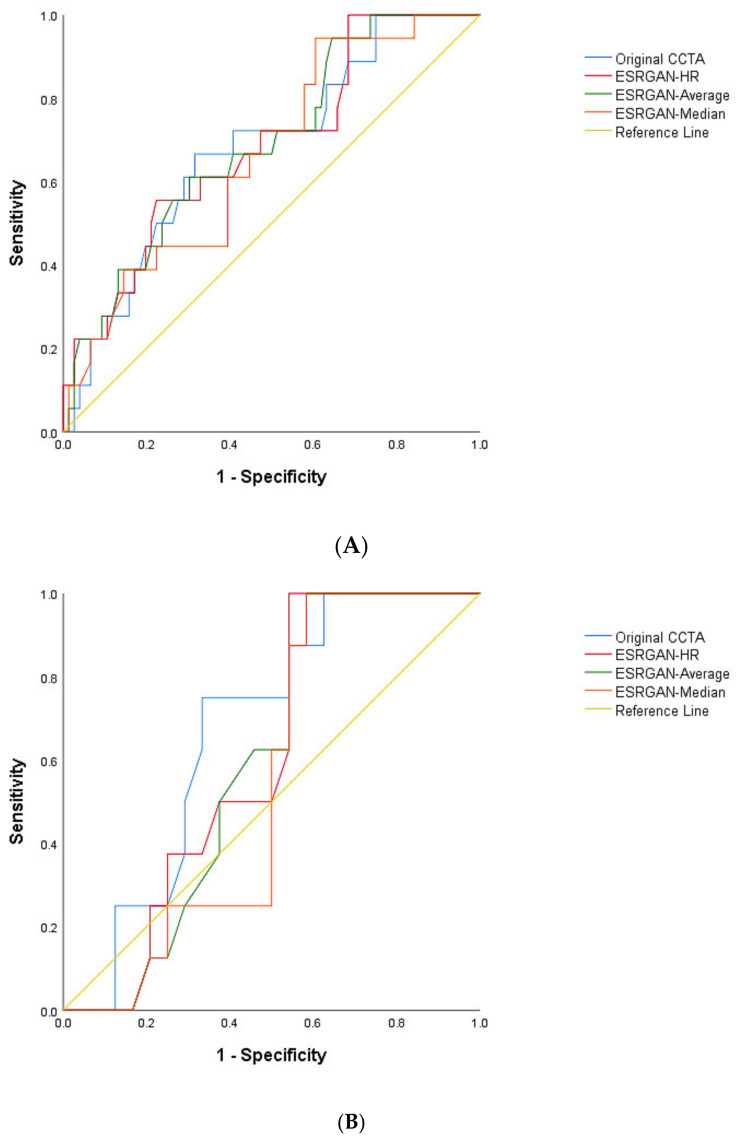

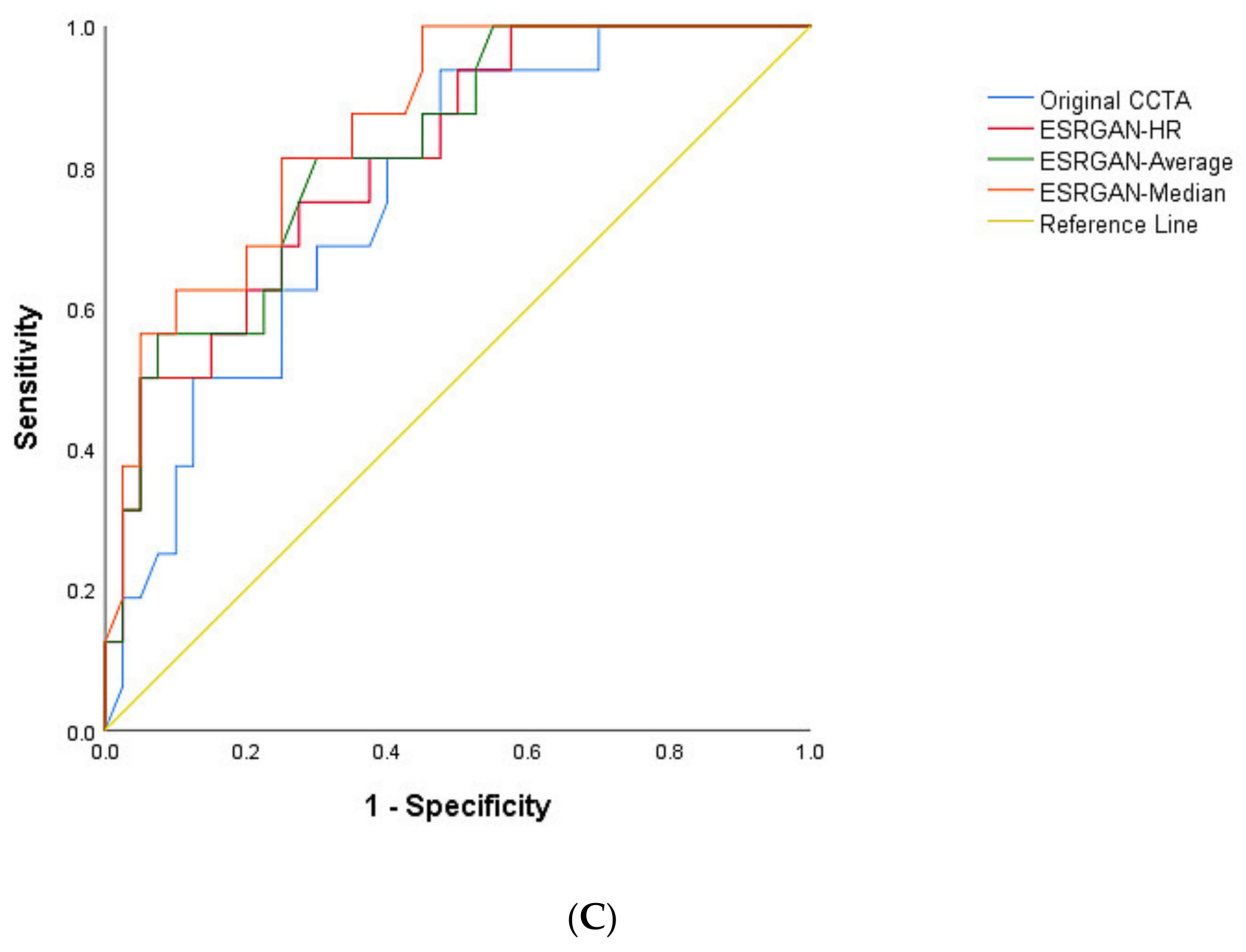
Area under the receiver operating characteristic curve (AUC) between original CCTA and ERSGAN-processed images at LAD, LCx and RCA (**A**–**C**). The AUC was the highest at the RCA level with value ranging from 0.81 to 0.86 for ESRGAN-processed images than the 0.76 for original CCTA images. CCTA-coronary computed tomography angiography; ESRGAN-enhanced super-resolution generative adversarial network; HR-high-resolution; LAD-left anterior descending; LCx-left circumflex; No.-number; RCA-right coronary artery.

The highest number of false positive rates was found in the original CCTA images resulting in the lowest specificity and PPV at LAD, LCx and RCA arteries as shown in Table 2. In contrast, the ESRGAN-processed images reduced the number of false positive rates when compared to the original CCTA images, with ESRGAN-Median approach having the most notable improvements in specificity, PPV, PLR and AUC at all of three coronary arteries (Table 2). The PPV was similar at the LAD and LCx between original CCTA and the ESRGAN-processed images, with only slight improvement, while the specificity was significantly increased at all three coronary artery levels with ESRGAN-processed images when compared to those with original CCTA images (Table 2). The AUC was the highest at the RCA than that at LAD and LCx (Figure 3).

**Figure 4:**
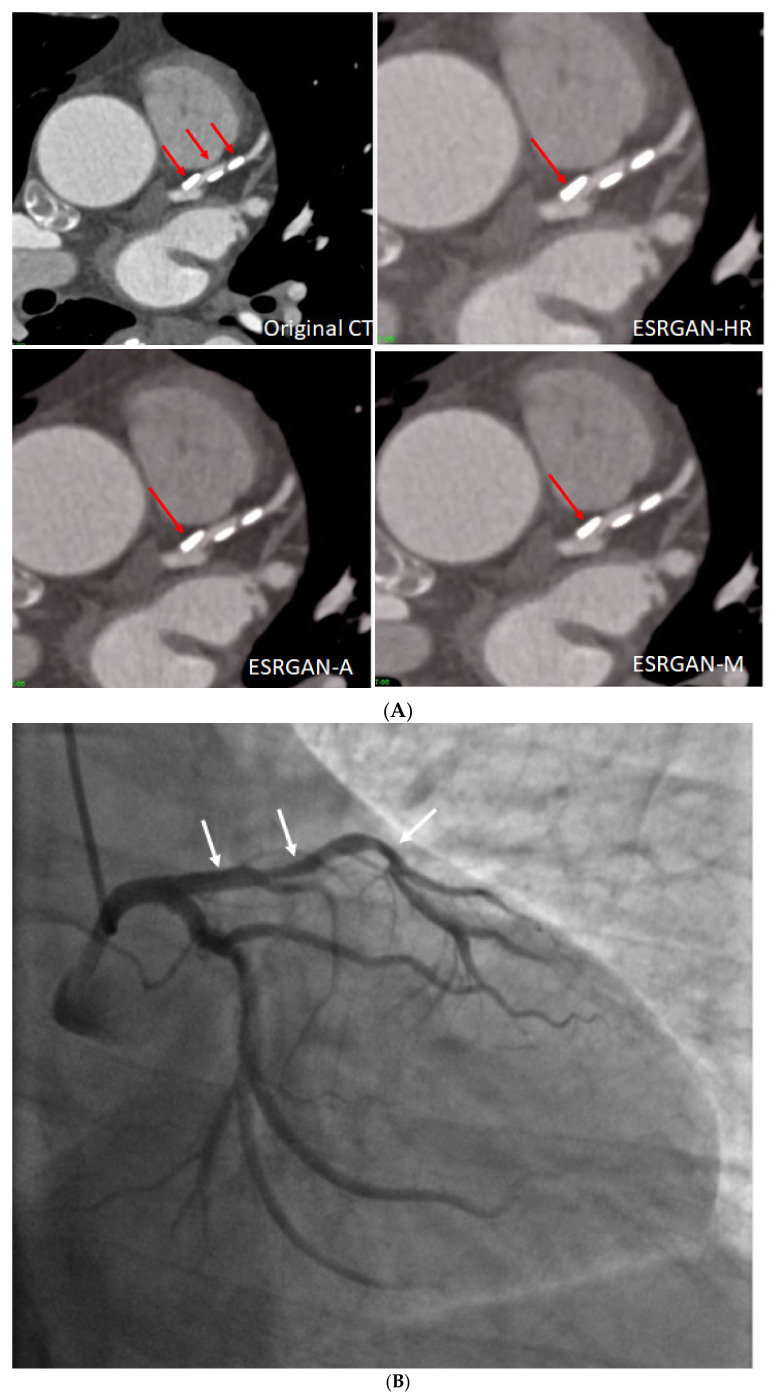
Multiple calcified plaques at the left anterior descending (LAD) artery in a 69-year-old man with coronary artery disease. (**A**): Comparison of the enhanced super-resolution generative adversarial network (ESRGAN)-processed images with the original coronary computed tomography angiography (CT) image in the assessment of coronary lumen stenosis caused by calcified plaques (arrows), with obvious improvement of lumen assessment, in particular, the ESRGAN-Median (ESRGAN-M) image in the assessment of the first plaque (long arrows). The measured mean stenosis was 63%, 44% and 86% at original CT, 58%, 39% and 83%, 56%, 37% and 83%, and 48%, 36% and 80% corresponding to ESRGAN-High Resolution (ESRGAN-HR), ESRGAN-Average (ESRGAN-A) and ESRGAN-Median (ESRGAN-M) images, respectively. (**B**): Invasive coronary angiography confirmed no significant stenosis at the LAD with corresponding diameters being 23%, 37% and 28% (arrows), respectively. ESRGAN-M images reduced the false positive rate by 33% when compared to the original CT and other two processed datasets.

**Figure 5:**
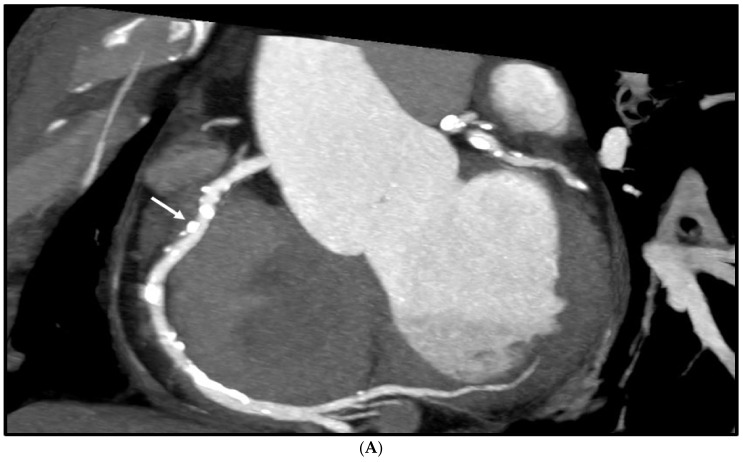

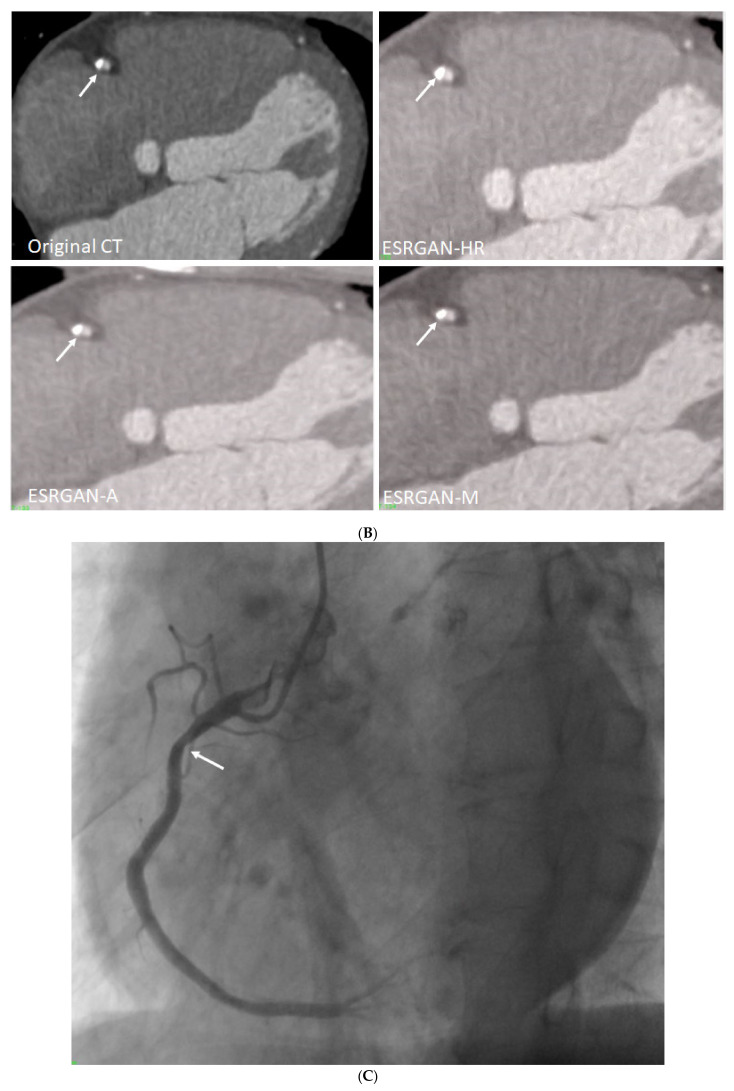
Multiple calcified plaques at the right coronary artery (RCA) in a 64-year-old man with coronary artery disease. (**A**): Curved planar reformatted image shows multiple calcified plaques at the RCA. Arrow refers to the plaque in the proximal RCA causing >50% stenosis that was selected for comparison of lumen differences. (**B**): Comparison of the enhanced super-resolution generative adversarial network (ESRGAN)-processed images with original CCTA in the assessment of coronary lumen stenosis caused by the plaque (arrows) as indicated in Figure 4A. The mean lumen stenosis was measured 59%, 52%, 48% and 47% at original CCTA, ESRGAN-High Resolution (ESRGAN-HR), ESRGAN-Average (ESRGAN-A) and ESRGAN-Median (ESRGAN-M) images, respectively. (**C**): Invasive coronary angiography confirms no significant stenosis with measured diameter of 33% (arrow). ESRGAN-A and M images improved the lumen assessment illustrating no significant stenosis as well.

Figure 4 is an example showing multiple calcified plaques at LAD with improvement in the visualisation of coronary lumen observed in ESRGAN-processed images when compared to the original images, while Figure 5 is another example demonstrating multiple calcified plaques at RCA showing improved lumen assessment with ESRGAN-processed images.

## 4. Discussion

This study tested the feasibility of using an advanced DL approach, ESRGAN to process the CCTA images with the aim of suppressing blooming artifact associated with heavy calcification in the coronary arteries. Our results show that ESRGAN-processed images improved coronary lumen visualisation with ESRGAN-Median approach resulting in up to 20% reduction of blooming artifact in the coronary lumen assessment. ESRGAN-Median processed images reduced the false positive rates by up to 33% when compared to the original CCTA images and this has significant clinical value with corresponding reduction of unnecessary invasive procedures, and potential of reclassification of patient risk assessment, and improvement in cost effectiveness regarding the judicious use of imaging modalities, in particular, reserving the downstream testing such as risky and costly ICA procedure only for patients with significant coronary stenosis.

It is a well-known fact that CCTA has limited diagnostic value in assessing calcified plaques with low specificity and PPV [3,4,6,7]. A number of traditional approaches or strategies have been developed to tackle this issue including the use of dual-energy CT [22,23], iterative reconstruction algorithms [24,25], high-resolution or high-definition CT [26,27], use of left coronary angulation [28,29,30] and conventional image processing or subtraction techniques [3,4,31]. Despite increased specificity and PPV to some extent, results are still not satisfactory due to applications either limited to research purpose, or lacking of sufficient sample size to draw robust conclusions, or the diagnostic value of CCTA not reaching the accuracy for patient’s diagnosis. Our previous studies using the same datasets along with other reports showed that the specificity and PPV ranged from 19% to 53%, with improved values just reaching around 70% through use of above-mentioned image processing methods [3,4,28,31]. Further, these previously used methods lacked efficiency and reliability. Thus, there is urgent need to develop advanced approaches for further improving the CCTA performance in calcified plaques with the potential to be applied to a wider patient population, and AI is a promising tool to fill the gap.

AI has become an increasingly used tool in the medical domain and its application in CAD including coronary stenosis and calcium scoring assessments has shown great promise. Studies have reported that AI, specifically, machine learning- (ML-) and DL-based CCTA image processing and analysis assists automatic segmentation and detection of coronary stenosis, with ML and DL-based risk assessment models improve risk stratifications and prediction of disease outcomes in patients with CAD [32,33]. These models allow quantification of coronary calcium deposits with similar or even better diagnostic accuracy than the traditional CCTA approach (manual detection and diagnosis of CAD) [34,35], and with significant reduction of post-processing and interpretation times by up to 85% [36,37]. Although these approaches improve image quality and image post-processing time through automatic quantification of calcium deposits, a paucity of research has been conducted on the use of DL to suppress the artifact associated with calcification in the coronary arteries. 

Recently, a study has reported the use of Canon AiCE reconstruction kernel to suppress the blooming artifact on the CCTA images. However, its findings show that the Canon AiCE reconstruction kernel could only achieve about 10% of PPV improvement for CCTA cases with 50% diameter stenosis or greater [38]. In contrast, our results (Table 2) illustrate that the use of ESRGAN could increase the specificity and PPV by at least 10% and up to 40%. Our superior results could be due to the use of the more recent DL method, GAN which was designed in 2014 while the Canon AiCE reconstruction kernel was developed based on the traditional method, CNN which emerged in 1980 [17,39]. For non-medical research, it has been demonstrated that GAN performs better than CNN for generating more realistic high-resolution images with unwanted noise removal which is essential for calcium deblooming [17,18]. Also, unlike the Canon AiCE reconstruction kernel which is vendor specific, our ESRGAN approach can be applied to any CCTA images [38].

Besides, our results show that the ESRGAN-processed images reduced the false positive rates, thus improving the specificity and PPV when compared to the original CCTA images. Among the three different ESRGAN-processed datasets, ESRGAN-Median shows the best outcomes. It is well known that the average and median (pixel) binning are common image processing techniques to suppress the image noise, thus improving coronary lumen visualisation for more accurate assessment [21,22]. However, the blooming artifact in CCTA is due to averaging two extreme values of attenuations of a high-density calcified plaque and a much-lower-density vessel, and hence using the median as the resultant pixel value would be a better representation of the actual structure, leading to the most accurate assessment [5].

Technological advancements in CT scanning techniques have enabled CCTA to quantitatively assess coronary plaques for risk classification and prediction of cardiac events. However, interpretation of CCTA images, in particular when characterizing high-risk coronary plaques depends on visual assessment and highly relies on a priori knowledge and experience of the reader (radiologist or cardiologist). It has been reported that the inter-observer reproducibility of assessing high-risk plaque features is poor even among experienced readers [40]. This limitation can be overcome with use of AI (ML) due to its ability of automatically extracting more complex imaging features from CCTA data [41,42,43]. Image interpretation bias and inter-observer variability were decreased when ML based on radiomics was incorporated into large CCTA datasets [42]. Combining DL with diameter assessment of coronary stenosis was found to significantly increase the diagnostic accuracy of CCTA in identifying significant stenosis when compared to using diameter assessment alone (AUC: 0.76 vs. 0.68) [44,45]. ML-based on radiomics represents a new image interpretation and analysis approach enabling more rapid extraction of large amount of clinical and imaging data, thus providing more accurate prediction of disease outcomes [46,47,48]. This could be the future research direction to solve the limited diagnostic value of CCTA in calcified plaques.

Our feasibility study shows promising results of improved diagnostic performance of CCTA with the use of ESRGAN model, however, there were some limitations that existed in this study. First, the sample size was small as we only analysed 50 cases with calcified plaques. This limitation was compensated by the analysis of 184 plaques, which was greater than that of the study about the use of Canon AiCE reconstruction kernel for calcium deblooming [38], although a greater sample size will be necessary in future studies. Second, the ESRGAN-processed images improved the specificity and PPV to 55% and 47%, however, these were still insufficient to meet diagnostic requirements in clinical practice. Usually, an imaging modality or technique with over 80–90% diagnostic value is deemed acceptable for use as a diagnostic approach, therefore, further improvement of the ESRGAN model is necessary to enhance its diagnostic value. This will be addressed in our ongoing study with use of an ESRGAN model finetuned by the CCTA images as Wang et al.’s [18] ESRGAN model was not trained by any medical images. Finally, this was a retrospective study with no patient follow-up as our purpose was to test the feasibility of ESRGAN for calcium deblooming. Once we achieve better results with the finetuned model, it will be tested on a large CCTA dataset with follow-up outcomes included, such as risk reclassification as a result of reduced blooming artifact and corresponding reduced number of unnecessary examinations, in addition to the diagnostic value evaluation. The eventual clinical utility of the developed AI-assisted calcium deblooming technique will make workflow more efficient, such as reducing the time taken for image interpretation and analysis by expert readers.

## 5. Conclusions

In conclusion, we tested the ESRGAN model for suppressing the blooming artifact in CCTA images of 50 cases with calcified plaques in the coronary arteries and our preliminary results show that the ESRGAN-processed images improved the specificity and PPV compared to the original CCTA images. The ESRGAN-processed images suppressed the blooming artifact associated with severe calcification in the coronary arteries, thus reducing the false positive rates. This has significant clinical value in improving the CCTA performance when assessing calcified plaques. Future research with the use of the finetuned ESRGAN model is needed to further enhance the diagnostic value of CCTA for patients with the calcified plaques.

## Figures and Tables

**Table 1:** Clinical characteristics of the study population.

Characteristics	Total Number of Cases (Percentage)
Age (years)	61.9 ± 9.1
M/F	41/9
Coronary artery involvement	
1-vessel disease	10 (20%)
2-vessel disease	28 (56%)
3-vessel disease	12 (24%)
Distribution of calcified plaques at coronary arteries	
LAD (*n* = 96)	
1–3 plaques	45 (90%)
4–5 plaques	5 (10%)
>5 plaques	0
LCx (*n* = 32)	
1–3 plaques	25 (100%)
4–5 plaques	0
>5 plaques	0
RCA (*n* = 56)	
1–3 plaques	19 (83%)
4–5 plaques	1 (4%)
>5 plaques	3 (13%)

F, female; LAD, left anterior descending artery; LCx, left circumflex artery; M, male; RCA, right coronary artery. AUC-area under the receiver operating characteristic curve; FN-false negative; FP-false positive; HR-high-resolution; LAD-left anterior descending artery; LCx-left circumflex artery; NLR-negative likelihood ratio; NPV-negative predictive value; No.-number; PLR-positive likelihood ratio; PPV-positive predictive value; RCA-right coronary artery; TN-true negative; TP-true positive.

**Table 2:** Diagnostic value of original coronary computed tomography angiography (CCTA) images and enhanced super-resolution generative adversarial network (ESRGAN)-processed images for assessment of calcified plaques on per-vessel assessment with invasive coronary angiography as the reference.

Coronary Arteries/No. Plaques	TP	FP	TN	FN	Sensitivity (%)	Specificity (%)	PPV (%)	NPV (%)	PLR	NLR	AUC
**LAD**											
Original CCTA	18	61	17	0	100 (81.5, 100)	21.8 (13.2, 32.6)	22.8 (20.8, 24.9)	100	1.28 (1.13, 1.43)	0.00	0.68 (0.55, 0.81)
ESRGAN-HR	18	54	24	0	100 (81.5, 100)	30.8 (20.8, 42.2)	25.0 (22.3, 27.9)	100	1.44 (1.24, 1.67)	0.00	0.69 (0.55, 0.82)
ESRGAN-Average	17	49	29	1	94.4 (72.7, 99.8)	37.2 (26.5, 48.9)	25.8 (22.1, 29.8)	96.7 (80.8, 99.5)	1.50 (1.22, 1.84)	0.15 (0.02, 1.02)	0.69 (0.56, 0.82)
ESRGAN-Median	17	46	32	1	94.4 (72.7, 99.8)	41.0 (30.0, 52.7)	26.9 (22.9, 31.4)	96.9 (82.4, 99.5)	1.60 (1.29, 1.99)	0.13 (0.02, 0.93)	0.66 (0.53, 0.79)
**LCx**											
Original CCTA	8	21	3	0	100 (63.1, 100)	12.5 (2.6, 32.4)	27.6 (24.7, 30.7)	100	1.14 (0.98, 1.33)	0.00	0.67 (0.48, 0.86)
ESRGAN-HR	8	18	6	0	100 (63.1, 100)	25.0 (9.8, 46.7)	30.8 (26.1, 35.9)	100	1.33 (1.06, 1.68)	0.00	0.61 (0.41, 0.80)
ESRGAN-Average	8	14	10	0	100 (63.1, 100)	41.7 (22.1, 63.4)	36.4 (28.9, 44.5)	100	1.71 (1.22, 2.40)	0.00	0.59 (0.40, 0.78)
ESRGAN-Median	8	14	10	0	100 (63.1, 100)	41.7 (22.1, 63.4)	36.4 (28.9, 44.5)	100	1.71 (1.22, 2.40)	0.00	0.55 (0.36, 0.75)
**RCA**											
Original CCTA	16	33	7	0	100 (79.4, 100)	17.5 (7.3, 32.8)	32.7 (29.6, 35.9)	100	1.21 (1.05, 1.39)	0.00	0.76 (0.64, 0.89)
ESRGAN-HR	16	23	17	0	100 (79.4, 100)	42.5 (27.0, 59.1)	41.0 (34.8, 47.6)	100	1.74 (1.33, 2.27)	0.00	0.81 (0.69, 0.93)
ESRGAN-Average	16	22	18	0	100 (79.4, 100)	45.0 (29.2, 61.5)	42.1 (35.5, 49.0)	100	1.82 (1.37, 2.41)	0.00	0.82 (0.71, 0.94)
ESRGAN-Median	16	18	22	0	100 (79.4, 100)	55.0 (38.5, 70.7)	47.1 (38.7, 55.6)	100	2.22 (1.58, 3.13)	0.00	0.86 (0.76, 0.96)

Numbers in the bracket indicate 95% confidence interval.

## Data Availability

The datasets used in this study are not publicly available due to strict requirements set out by authorised investigators.
